# The effectiveness of percutaneous endoscopic decompression compared with open decompression and fusion for lumbar spinal stenosis: protocol for a multicenter, prospective, cohort study

**DOI:** 10.1186/s12891-022-05440-4

**Published:** 2022-05-27

**Authors:** Shuheng Zhai, Wenkui Zhao, Bin Zhu, Xin Huang, Chen Liang, Bao Hai, Lixiang Ding, Hongwei Zhu, Xianhai Wang, Feng Wei, Hongling Chu, Xiaoguang Liu

**Affiliations:** 1 Department of Orthopedics, Peking University Third Hospital, Beijing, China; 2 Engineering Research Center of Bone and Joint Precision Medicine, Beijing, China; 3 Beijing Key Laboratory of Spinal Disease Research, Beijing, China; 4 Pain Medicine Center, Peking University Third Hospital, Beijing, China; 5 Department of Orthopaedics, Capital Medical University Affiliated Beijing Friendship Hospital, Beijing, China; 6 Department of Orthopedics, Capital Medical University Affiliated Beijing Shijitan Hospital, Beijing, China; 7 Department of Neurosurgery, Xuanwu Hospital Capital Medical University, Beijing, China; 8 Department of Orthopedics, Beijing Changping Hospital, Beijing, China; 9 Research Center of Clinical Epidemiology, Peking University Third Hospital, 49 North Garden Road, Haidian District, Beijing, 100191 China

**Keywords:** Lumbar spinal stenosis, Percutaneous endoscopic decompression, Open decompression and fusion, Comparative effectiveness

## Abstract

**Background:**

Lumbar spinal stenosis (LSS) is one of the most frequent indications for spine surgery. Open decompression and fusion surgery was the most common treatment and used to be regarded as the golden standard treatment for LSS. In recent years, percutaneous endoscopic decompression surgery was also used for LSS. However, the effectiveness and safety of percutaneous endoscopic decompression in the treatment of LSS have not been supported by high-level evidence. Our aim is to 1) compare the effectiveness of percutaneous endoscopic decompression surgery and open decompression and fusion for the treatment of LSS. 2) Investigate the prognosis risk factors for LSS. 3) Evaluate the influence of percutaneous endoscopic decompression for the stability of operative level, and degeneration of adjacent level.

**Methods:**

It’s a prospective, multicenter cohort study. The study is performed at 4 centers in Beijing. This study plans to enroll 600 LSS patients (300 patients in the percutaneous endoscopic decompression group, and 300 patients in the open decompression and fusion group). The demographic variables, healthcare variables, symptom related variables, clinical assessment (Visual analogue score (VAS), Oswestry disability index (ODI), Japanese Orthopaedic Association score (JOA)), and radiological assessment (dynamic X-ray, CT, MRI) will be collected at baseline visit. Patients will follow up at 3, 6, 12 months. The primary outcome is the difference of improvement of ODI between baseline and 12-month follow-up between the two groups. The secondary outcome is the score changes of preoperative and postoperative VAS, the recovery rate of JOA, MacNab criteria, patient satisfaction, degeneration grade of adjacent level, ROM of operative level and adjacent level, complication rate.

**Discussion:**

In this study, we propose to conduct a prospective registry study to address the major controversies of LSS decompression under percutaneous spinal endoscopy, and investigate the clinical efficacy and safety of percutaneous endoscopic decompression and open decompression in the treatment of LSS.

**Trial registration:**

This study has been registered on clinicaltrials.gov in January 15, 2020 (NCT04254757). (SPIRIT 2a).

## Background

Lumbar spinal stenosis (LSS) is caused by the degeneration of the lumbar spine including disc herniation, the hypertrophy of facet joint and ligamentum flavum, and the formation of osteophyte [1]. The characteristics of LSS are low back pain, sciatica, and intermittent claudication which will seriously affect the daily life and work of patients [1, 2].

LSS is the most frequent indication for spine surgery among the elderly population [3, 4], and previous studies have demonstrated that surgical treatment was better than conservative treatment in selected patients [5, 6].

Open decompression and fusion was the most common treatment and used to be regarded as the golden standard treatment for LSS [7, 8]. However, due to the surgical trauma caused by open decompression and fusion surgery, complications, and the acceleration of adjacent level degradation, more minimally invasive treatments were required [9–11].

Percutaneous endoscopic decompression is a minimally invasive treatment which has the advantage of operated under local anesthesia, less surgical trauma, fewer complications, shorter hospital stay, and rapid postoperative recovery. The application of percutaneous endoscopic decompression has been limited to soft disc herniation. In recent years, with the development of endoscopic instruments and surgical techniques, some surgeons applied percutaneous endoscopic decompression for the treatment of LSS [12–14]. However, the related studies had some limitations including small sample size, short-term follow-up, single-center, no control group, retrospective study which reduced the quality of evidence for the conclusion. (SPIRIT 6a,b).

The present prospective, multicenter, large sample size cohort study based on real-world will be helpful for the evaluation of the clinical effectiveness and safety of the percutaneous endoscopic decompression for LSS. Furthermore, we designed to analyze various influence factors for the prognosis of LSS including degeneration grade, pathology type, stenosis grade, decompression range. The present study will provide useful information for the development of guidelines and standards for percutaneous endoscopic decompression for the treatment of LSS.

## Methods

### Aim and objective

The primary aim of this study is to compare the effectiveness and safety of percutaneous endoscopic decompression surgery and open decompression and fusion surgery for the treatment of patients with LSS in the real-world treatment setting. The secondary aim is to investigate the prognosis factors for LSS treated by surgery.

The objectives of this study:

1. Compare the clinical outcomes of percutaneous endoscopic decompression and open decompression and fusion for lumbar spinal stenosis.
2. Investigate the influence of various factors (including demographic variables, risk factors, treatment factors, and radiological assessment) for the clinical outcome of LSS treated by surgery.
3. Investigate the influence of percutaneous endoscopic decompression surgery on stability of the operated level.
4. Investigate the influence of percutaneous endoscopic decompression surgery on degeneration of adjacent level. (SPIRIT 7)

### Study design

The present study is a multicenter, prospective, cohort study that will compare the effectiveness of percutaneous endoscopic decompression surgery and open decompression and fusion surgery for lumbar spinal stenosis in the real-world treatment setting. The study will enroll 600 patients (300 patients in the percutaneous endoscopic decompression group, and 300 patients in the open decompression and fusion group) from 11 June 2020 to 31 December 2022. The patient will be followed up at 3, 6, and 12 months postoperatively. The study is due to be completed on 31 December 2022. (SPIRIT 8).

### Sample size calculation

The sample size calculation was based on the ODI score at 12 months follow-up. According to the retrospective study of our center and previous literature reported [15], the mean value and standard deviation of ODI score in 12 months after surgery was 20 and 15 in the percutaneous endoscopic decompression group, and 15 and 10 in the open decompression and fusion group. Assuming an overall type 1 error (α) of 0.05 and type II error (β) of 0.1, we needed 137 patients in each group to detect the difference of ODI in two groups. Considering the final follow-up rate of 80% and the center effect value of 1.2, a minimum of 206 patients were needed in each group. Moreover, this study is a real-world observation study, it is necessary to consider confounding factors when analyzing the results. We estimated that 300 patients in each group will be enrolled in this study. (SPIRIT 14).

### Study population and enrollment

**Fig. 1:**
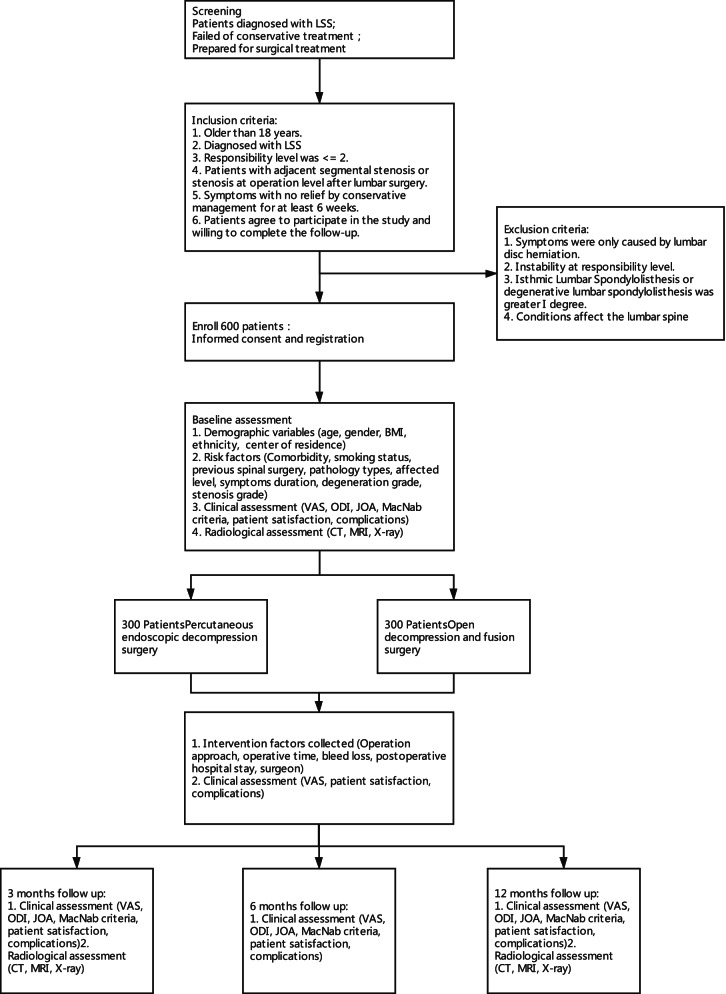
Flow chart of the study. VAS, Visual analogue score (VAS); ODI, Oswestry disability index; JOA, Japanese Orthopaedic Association score. X-ray including anterior-posterior position, lateral position, flexion and extension position lumbar X-ray

The target population of this study was patients diagnosed with LSS who failed of conservative treatment and prepared for surgical treatment. The study is performed at Peking University Third Hospital, Beijing Shijitan Hospital of capital medical university, Xuanwu Hospital of capital medical university, and Beijing Changping Hospital. Potential participants will be screened by experienced orthopaedic surgeons or neurosurgeons at each clinical center. Patients met the eligibility criteria will be offered the detailed description of all alternative treatment. After careful consideration, patients will be asked to select their preferred treatment approach. When patients decide upon their treatment option, the physicians will explain this protocol and informed consent to participate will be obtained. All procedures of the study, and the rights, responsibilities, benefits, risks of the participant will be completely informed, and the informed consent form will be signed. Flow chart of the study design is shown in Fig. 1. (SPIRIT 9, 15).

### Eligibility criteria

#### Inclusion criteria

1. Older than 18 years.
2. Diagnosed with lumbar spinal stenosis.
3. Responsibility level was <= 2.
4. Patients with adjacent segmental stenosis or stenosis at operation level after lumbar surgery.
5. Symptoms with no relief by conservative management for at least 6 weeks.
6. Patients agree to participate in the study and willing to complete the follow-up.

#### Exclusion criteria

1. Symptoms were only caused by lumbar disc herniation.
2. Instability at responsibility level.
3. Isthmic Lumbar Spondylolisthesis or degenerative lumbar spondylolisthesis was greater I degree.
4. Conditions affect the lumbar spine (infection, tumor, and neurological diseases, etc.) (SPIRIT 10)

### Treatment

#### Percutaneous endoscopic decompression group

##### Transforaminal approach

Patients were placed in a lateral position. The surgery was performed under local anesthesia (1% Ropivacaine 10 ml, 2% Lidocaine 15 ml, 0.9% Normal Saline 20 ml). After confirmed the entry point and the direction of approach, an 18-guage spinal needle was inserted to the surface of the superior articular process by the guidance of fluoroscopy. Insert a guidewire to replace the spinal needle, and then a series obturator was inserted through the guidewire. Finally, a working cannula was inserted through the obturator. After the location of the cannula was confirmed by fluoroscopy, the endoscope system and endoscopic trephine were inserted. The foramen, lateral recesses, and the posterior of the vertebral body could be decompressed by the transforaminal approach.

##### Interlaminar approach

Patients were placed in a prone position. The surgery was performed under 5μg Sufentanil intravenously and local anesthesia (1% Ropivacaine 10 ml, 2% Lidocaine 15 ml, 0.9% Normal Saline 20 ml). Inserted an 18-guage spinal needle to the posterior of ligamentum flavum, and confirmed the needle is located at the midpoint of the interlaminar space by the fluoroscope. Inserted the guidewire, obturator, working cannula, endoscopic trephine, and the endoscope system in sequence. The foramen, lateral recesses, and central canal could be decompressed by the interlaminar approach. The unilateral decompression or bilateral decompression could be chosen depending on the stenosis.

All decompression procedures of both approaches were performed under the observation of the endoscopic view. The decompression was completed when compressed dura and nerve root could be seen, and the nerve root could be mobilized freely by a flexible probe.

#### Open decompression and fusion group

Patients were placed in a prone position. The surgery was performed under general anesthesia. After exposed to the lamina, inserted the pedicle screw. The posterior decompression including laminectomy, lateral recesses resection, and foraminotomy was performed according to the stenosis. Interbody fusion or posterolateral fusion is determined by the surgeon’s preference.

‘X’ indicates that the procedure is carried out.

VAS, Visual analogue score (VAS); ODI, Oswestry disability index; JOA, Japanese Orthopaedic Association score. X-ray including anterior-posterior position, lateral position, flexion and extension position lumbar X-ray.

### Follow up

Patients will be followed up at 3, 6, 12 months after surgery. Clinical and radiological assessment at 3 and 12 months after surgery will be performed through outpatient follow-up. Clinical assessment at 6 months after surgery will be completed through email or telephone (Table 1). Adverse events will be recorded during the follow-up period. (SPIRIT 13, 18b).

**Table 1:** Data collection

Variables	Baseline	Perioperative	3 months	6 months	12 months
Demographic Variable					
Age	X				
Gender	X				
BMI	X				
Ethnicity	X				
Center of residence	X				
Risk Factors					
Comorbidity	X				
Smoking status	X				
Previous spinal surgery	X				
Pathology types	X				
Affected level	X				
Symptoms duration	X				
Degeneration grade	X				
Stenosis grade	X				
Treatment Factors					
Operation approach		X			
Operative time		X			
Bleed loss		X			
Postoperative hospital stay		X			
Surgeon		X			
Radiological Assessment					
X-ray	X		X		X
CT	X		X		X
MRI	X		X		X
Clinical Assessment					
VAS (back & leg)	X	X	X	X	X
ODI	X		X	X	X
JOA	X		X	X	X
MacNab criteria			X	X	X
Patient satisfaction		X	X	X	X
Complications		X	X	X	X

### Measures

#### Exposure measures

The exposure measures include demographic variables, risk factors and treatment factors. The demographic variables included age, gender, BMI, ethnicity, and center of residence. The risk factors were composed of healthcare variables (Comorbidity, smoking status, previous spinal surgery), and symptom related variables (Pathology types, affected level, symptoms duration, degeneration grade, stenosis grade). The treatment factors included operation approach, operative time, bleed loss, postoperative hospital stay, surgeon. The demographic variables, and risk factors were collected after patients were enrolled in the study, and before the surgery was performed. The treatment factors were collected before patients were discharged. (Table 1).

#### Special measures

The special measures were composed of clinical assessment and radiological assessment.

### Clinical assessment

Visual analogue score (VAS) [16], Oswestry disability index (ODI) [17], Japanese Orthopaedic Association score (JOA) [18], MacNab criteria, Patient satisfaction, Complications.

VAS divided the degree of pain from 0 to 10. 0 represented no pain, and 10 represented the most pain. VAS was used to evaluate the pain of the back and lower limb separately.

ODI was wildly used to evaluate the limitations of daily activities caused by spinal diseases. The simplified Chinese version ODI was composed of 10 questions, including pain intensity, self-care, lifting, walking, sitting, standing, sleep, sex, social, travel. The score range for each question was 0–5 points. Summarized each question patient answered and converted into a percentage score.

JOA was an effective method to assess lumbar diseases and the recovery of lumbar spine surgery. The simplified Chinese version JOA included five aspects: low back pain (four items), lumbar function (six items), walking ability (five items), social life function (four items), and mental health (seven items). The recovery rate was calculated according to preoperative and postoperative JOA.

MacNab criteria were used to access the overall effectiveness of the surgery, including four grades: excellent, good, fair, poor.

Patient satisfaction was the patient’s subjective impression of surgery, including three grades: satisfied and accept, not satisfied but accept, not satisfied, and not accept.

### Radiological assessment

Radiological assessment included CT, MRI, anterior-posterior position, lateral position, flexion and extension position lumbar X-ray.

The lumbar spinal stenosis grade of central canal stenosis [19], lateral recess stenosis [20], and foraminal stenosis [21] was evaluated on MRI. The stenosis grade was divided into mild, moderate, and severe.

The degeneration grade of intervertebral disc of operative level and adjacent level was accessed on MRI according to Pfirrmann grade [22].

The decompression range (including the maximum sagittal diameter of the axial spinal canal, lateral recess angle, lateral recess sagittal diameter, disc height, and volume of bone resected, and etc.) was measured on CT [23, 24].

The range of motion (ROM) of operative level and adjacent level was measured by the Cobb angle of function spinal unit (FSU) on flexion and extension position X-ray [25].

### Outcome measures

#### Primary outcome measure

The primary outcome measure is the score changes of ODI between baseline and 12-month follow-up.

#### Secondary outcome measures

The secondary outcome measures are:

1. The score changes between baseline and 3 months, 6 months, 12 months follow up in: JOA, and VAS.
2. The MacNab criteria of 3 months, 6 months, 12 months follow up. The complication rate and patient satisfaction rate before discharged, and at 3 months, 6 months, 12 months follow up.
3. The changes between baseline and 3 months, 6 months, 12 months follow up in: degeneration grade of adjacent level, stenosis grade of operative level, ROM of operative level and adjacent level.
4. The relationship between the exposure measures, special measures and the postoperative clinical outcomes. (SPIRIT 12)

### Data collection and management

The case report form (CRF) designed base on study protocol will be used to record the data. Data collection is performed by trained researchers in accordance with the standardized processing. An electronic research database was established on Research Manager (ResMan) to manage the data. Data entry will be performed by one researcher, and data verification will be carried out by another researcher. (SPIRIT 19).

### Quality control

Every researcher participated in this study will be trained in terms of protocol, CRF, data collection, clinical assessment, and radiological assessment. All radiological data will be measured by two researchers independently, and the final outcome was the average of their measures. The data with major difference between their measures will be measured again, and explain the reason. After the study completed, all data will be checked again. Then the database will be locked after all data are checked and confirmed,. All the original data files will be stored for a period according to corresponding regulations. (SPIRIT 18a).

### Patient and public involvement

Patient and public did not participate in the design, recruitment, conduction of this study.

### Statistical analysis

The demographic characteristics, prognosis factors and treatment factors will be described by using the general statistical description method according to the distribution of the data. Difference in difference analysis of ODI will be used to compare the effectiveness of percutaneous endoscopic decompression group with open decompression and fusion for lumbar spinal stenosis group. The multivariable analysis will be conduct to determine the effect size of treatment factors and potential prognosis factors on outcome. Subgroup analysis will be also conducted to compare the clinical effectiveness of open surgery and endoscopic decompression in patients with different degeneration grades, age, and approaches.

All statistical analyses were conducted using SPSS 25.0 (USA). Statistical significance was accepted at a *p* value of less than 0.05. (SPIRIT 20a).

### Missing data

At the final complete analysis for the primary outcome, we will exclude the cases which missing ODI data at 12 months follow-up [26]. Missing data could influence the validity of prospective studies, we will aim to maximize the follow-up rate to above 80%. (SPIRIT 20c).

### Ethics and dissemination

This study has been approved by the Hospital’s Medical Science Research Ethics Committee (IRB00006761-M2020022). All participants will provide written informed consent and could withdraw from the study at any time. The results of this study will be disseminated by peer-reviewed and open access publications.

## Discussion

The goal of this study is to evaluate the real-world effectiveness and safety of percutaneous endoscopic decompression surgery versus open decompression and fusion surgery for LSS. Moreover, we also planned to investigate the influence of various factor on clinical outcomes of LSS.

Percutaneous endoscopic decompression is an advanced mini-invasive technique. Sun et al. [14] introduced an interlaminar approach endoscopic decompression technique to treat lumbar central canal stenosis, the primary outcomes of 38 patients was satisfying.

Lee et al. [12] analyzed 213 patients with lumbar canal stenosis or lateral recess stenosis treated by percutaneous endoscopic decompression, the outcomes of 26 months follow-up showed the satisfied rate reached to 93.8%.

However, there have been some concerns about the application of the percutaneous endoscopic decompression for LSS. The first concern is whether the percutaneous endoscopic decompression could provide sufficient decompression without affecting the stability of the operative level. Secondly, whether the percutaneous endoscopic decompression prevents the degeneration of adjacent level. Finally, the recurrence of symptoms its influence factors.

In this study, the dynamic X-ray, CT, and MRI were performed preoperatively and at 3, 12 months follow-up. The instability of operative level will be assessed according to the changes of ROM and VAS (back) [25]. The decompression range and the degeneration grade will be also measured. These will clarify the exact influence of percutaneous endoscopic decompression on the stability of operative level and the degeneration of adjacent level. Furthermore, various influence factors including demographic variables, healthcare variables, symptom related variables, treatment factors, degeneration grade, stenosis grade, decompression range, stability of operative level, and degeneration of adjacent levels were evaluated. The relationship between these various influencing factors and clinical effectiveness will be helpful to solve the controversial issues of percutaneous endoscopic decompression treatment for LSS.

To the best of our knowledge, this is the first prospective, multicenter, large sample study comparing endoscopic decompression and open decompression fusion treatment for LSS which incorporated clinical evaluation, dynamic X-ray, CT, and MRI.

### Limitation

The main limitation of this study is that, treatment group in this observational study is clinical routine treatment determined by the shared-decision making between patient and doctor, rather than randomization, which might lead to selection bias. However, the results could be strengthened by the well-designed prospective study, multicenter, large sample size, and the specific inclusion and exclusion criteria [27], and potential confounding factors will be collected for data analysis to minimize the impact of the selection bias.

### Trial status

The protocol was the second version (V2, 2020/06/30). The protocol status was in recruiting status and have not completed, in which the recruitment began on 11 June 2020 and it will be completed on 31 December 2022. (SPIRIT 3).

## Data Availability

Not applicable.

## Abbreviations

LSS: Lumbar spinal stenosis
VAS: Visual analogue score
ODI: Oswestry disability index
JOA: Japanese Orthopaedic Association score
ROM: range of motion
FSU: function spinal unit
CRF: case report form
ResMan: Research Manager
